# The Influence of Polyvinyl Alcohol Porogen Addition on the Nanostructural Characteristics of Hydroxyapatite

**DOI:** 10.3390/ma16186313

**Published:** 2023-09-20

**Authors:** Indrika Novella, Bedah Rupaedah, Diana Rakhmawaty Eddy, Suryana, Ferli Septi Irwansyah, Atiek Rostika Noviyanti

**Affiliations:** 1 Department of Chemistry, Faculty of Mathematics and Natural Sciences, Padjajaran University, Jl. Raya Bandung-Sumedang Km. 21, Bandung 45363, Indonesia; indrika21001@mail.unpad.ac.id (I.N.); diana.rahmawati@unpad.ac.id (D.R.E.); ferli@uinsgd.ac.id (F.S.I.); 2 Research Center for Applied Microbiology, National Research and Innovation Agency, Jl. Raya Jakarta-Bogor Km. 46, Bogor 16911, Indonesia; beda001@brin.go.id; 3 Department of Biology, Faculty of Mathematics and Natural Sciences, Padjajaran University, Jl. Raya Bandung-Sumedang Km. 21, Bandung 45363, Indonesia; suryana.bio@unpad.ac.id; 4 Department of Chemistry Education, UIN Sunan Gunung Djati, Bandung Jl. A.H. Nasution No. 105, Bandung 40614, Indonesia

**Keywords:** hydroxyapatite, polyvinyl alcohol, porogen

## Abstract

Hydroxyapatite (HA) is a porous material widely developed in various research fields because of its high biodegradability, biocompatibility, and low toxicity. In this research, HA was synthesized using a hydrothermal method with chicken eggshells as a calcium source and various concentrations of polyvinyl alcohol as a porogen (2.5%, 5.0%, and 7.5% by wt). The structure and morphology of HA were determined by X-ray diffraction (XRD) and scanning electron microscope (SEM), respectively. HA was obtained with varying concentrations of polyvinyl alcohol (PVA) porogen according to Inorganic Crystal Structure Database (ICSD) standard. Based on analysis using a refinement method, changes in unit cell parameters (cell volume and lattice strain) of HA synthesized using PVA porogen compared to the standard, the chi square (χ^2^) and index of *R* values were relatively low, validating the acceptable of the data. In addition, HA [Ca_10_(PO_4_)_6_(OH)_2_] with hexagonal structure and the P63/m space group was successfully obtained. Morphological analysis of HA by SEM found that HA has a spherical shape, and the porosity of HA increases with increasing concentrations of polyvinyl alcohol. The highest porosity was obtained with an addition of 5.0 wt% of PVA porogen (HAP3), reaching 69.53%.

## 1. Introduction

Hydroxyapatite (HA), with the chemical formula Ca_10_(PO_4_)_6_(OH)_2_, is a biomaterial that has garnered significant attention in recent years due to its desirable properties, such as high biodegradability and biocompatibility. Additionally, hydroxyapatite can be resorbed in a physiological environment. It is non-toxic and non-immunogenic [1,2]. The most commonly encountered crystal structure of HAs is hexagonal with the space group P*63/m* [3]. In addition, HA is found in another structure, namely monoclinic, with P*21/b* space group. The most common difference in hexagonal and monoclinic structures is the orientation of hydroxyl groups [4]. HA has numerous applications in various fields, including as a nanocarrier for drug delivery [4], environmental remediation [5], and enzyme immobilization [6]. 

Hydroxyapatite (HA) as a biomaterial for various applications requiring interaction with different biological tissues in living organisms has been discussed. Due to its biocompatibility and physicochemical similarity, HA has been examined as a material for various applications including bone implants in humans, dental adhesive materials, and bacterial carriers [7]. HA has also been used as a carrier for controlled release of compounds because it is the most stable calcium phosphate at ambient temperature and under physiological conditions. The synthesis procedure of nano HA significantly influences the shape, crystal structure, and phase integrity of hydroxyapatite particles, ultimately governing the mechanical characteristics of materials designed for biomedical purposes [8]. In drug delivery systems, HA has also been used for controlled and targeted drug release with enhanced adsorption across biological barriers [9]. The utilization of HA in these applications often requires specific pore designs, where the porosity needs to be optimized for specific applications. This has led to the development of various synthesis processes for modifying the porosity of hydroxyapatite biomaterials and it can be controlled and determined based on the fabrication methods. Various methods have been used for the synthesis of HA, such as hydrothermal [10], precipitation [11], solid state reaction [12], and sol-gel methods [13]. The hydrothermal method is one of the most widely used methods for synthesis material, including HA. Since this method also has good variable control of temperature, pressure, and composition/precursor, it can also be used for almost all forms of material, such as single crystals, nanoparticles, films, powders, fibers, polymers, and also ceramics [14]. In addition, the hydrothermal method is a simple method. Its ease of use also offers good control over material morphology and size [2]. Through the hydrothermal technique, hydroxyapatite (HA) nanopowder has been effectively synthesized, offering potential applications in both biomedical contexts and scientific investigations. This successful synthesis method opens avenues for utilizing the HA nanopowder in various biomedical applications and serves as a valuable resource for scientific research endeavors [15]. 

Increasing the porosity of hydroxyapatite will enhance its functionality by allowing its interactions with other substances to be more pronounced. Due to its similarity to natural bone mineral and its biocompatibility, the functionalization of hydroxyapatite can be tailored with suitable properties for various applications, such as drug delivery, tissue engineering, and bone regeneration. Processes to enhance the applications of porous hydroxyapatite include surface modification, bioactive molecules [15], loading and release of drugs [1], surface coatings [16], electrochemical functionalization [3], polymer coatings, metal ion doping [17], and photocatalytic functionalization [18]. The selection of the functionalization approach relies on the intended usage and the particular properties that are needed. The method chosen for functionalization is contingent upon the specific requirements and goals of the application. The functionalization technique to be employed is determined by the desired application and the distinct properties that are necessary for it. The decision regarding the method of functionalization is guided by the intended purpose and the specific properties that are deemed essential. The design and characterization of hydroxyapatite must be conducted carefully to ensure its effectiveness and safety for the intended purposes. The addition of a porogen is commonly carried out to enhance the porosity of a material, including hydroxyapatite.

A porogenic agent, or porogen, one of the components responsible for pore formation, played a crucial role in achieving controlled characteristics of HA pores [19]. Porogen can be in the form of crystals, particles, or fibers that readily evaporate or dissolve, such as paraffin, starch, sucrose, cellulose derivatives, water soluble polymers such as polyvinyl alcohol (PVA), polyvinyl pyrrolidone (PVP), and other polymers. Material porosity typically occurs due to the release of easily removable compounds, leaving pores behind [20]. PVA exhibits many versatile physicochemical properties; therefore, PVA is used in various fields of research. PVA is widely used as a porogenic agent for material pore modification because it is a biocompatible polymer, soluble in water, and it is frequently employed in synthesizing biomaterials due to its non-toxic nature. This polymer has a low melting point compared to the densification temperature of HA, allowing it to evaporate during the sintering process and create pores or cavities in HA nanoparticles [21,22].

Nowadays, the utilization of porogenic agents, especially polymers for designing the porosity of HA, has been widely explored. For instance, carbon has been used as a porogen, affecting the microstructure and enhancing the porosity of calcium phosphate granules [23]. Polyvinyl alcohol (PVA), polyvinyl pyrrolidone (PVP), and polyethylene oxide (PEO) have also been employed as a porogen in the fabrication process of HA scaffolds synthesized from golden snail shells as precursors. The porogen concentrations used are 0, 5, 10, and 15 wt%; however, the outcome is HA that exhibits higher agglomeration and high impurities due to an excessive porogen concentration. Therefore, in this study, lower porogen concentrations are utilized [21]. Paraffin has also been used as a porogenic agent in synthesizing porous HA scaffolds for bone tissue engineering applications [24]. 

The effect of porogen concentration on the properties of hydroxyapatite will significantly influence the porosity and structure of the resulting material. When porogens are added during the fabrication process of hydroxyapatite, they create voids or pores within the material which are subsequently removed. This porosity can have a notable impact on the characteristics and performance of the material, including porosity, surface area and osteoconductivity [25], mechanical properties and cell interactions [26], also drug delivery [27]. Control and optimization of porogen concentration can enable the customization of hydroxyapatite properties to meet desired outcomes for specific applications.

The focus of the present study was on the use of a hydrothermal method to investigate the influence of various concentrations (0, 2.5%, 5.0%, and 7.5% by wt%) of PVA as a porogen on the structural characteristics and porosity of HA synthesized from utilization of eggshell waste.

## 2. Materials and Methods

In this research, there are four main stages, including the isolation of calcium oxide from chicken eggshells as a calcium source, the synthesis of porous hydroxyapatite (HA) with the addition of polyvinyl alcohol (PVA) porogen, and the characterization of the structure and morphology of porous HA.

### 2.1. Materials

The chicken eggshells were collected from the Jatinangor region (utilization chicken eggs) as a source of calcium, along with diammonium hydrogen phosphate (Merck, Germany), polyvinyl alcohol, and distilled water.

### 2.2. Methods

#### 2.2.1. Calcium Preparation

Calcium was isolated from chicken eggshells, where the eggshells were cleaned of any remaining debris and membranes and then dried. The dried eggshells were ground using a ball mill and sieved through a 100-mesh sieve to obtain a powder. The eggshell powder was then calcined for 5 h at 1000 °C, producing white calcium powder [28].

#### 2.2.2. Hydroxyapatite Synthesis

Hydroxyapatite (HA) synthesis was based on previous research [21,28]. A total of 2.7925 g of calcium oxide and 3.9663 g of diammonium phosphate with a molar ratio of Ca/P = 1.67 were dissolved in 50 mL of distilled water. The mixture was then transferred to an autoclave and heated at 230 °C for 48 h. The resulting precipitate was filtered, washed with distilled water until reaching a pH of 7, and dried at 110 °C for 2 h. Next, the obtained HA powder was modified using polyvinyl alcohol (PVA) porogen, the concentration of PVA porogen was calculated based on Equation (1) [24].

(1) $$\mathrm{wt}\%\;\mathrm{of}\;\mathrm{polyvinyl}\;\mathrm{alcohol}\;(\mathrm{PVA})=\frac{\mathrm{PVA}\;\mathrm{mass}}{\mathrm{PVA}\;\mathrm{mass}+\mathrm{HA}\;\mathrm{mass}}$$

Varying concentrations of polyvinyl alcohol (PVA) solution (0%, 2.5%, 5.0%, and 7.5% by *wt*) were prepared by dissolving PVA in 50 mL of distilled water and heating it at 125 °C for 2 h. The PVA solution was mixed with the HA powder and stirred until homogeneous. The mixture was subsequently dried at 100 °C for 2 h, and the resulting dried precipitate was sintered at 1000 °C for 6 h. The synthesized hydroxyapatite samples were labeled as HAP1 (without porogen addition), HAP2 (2.5 wt%), HAP3 (5.0 wt%), and HAP4 (7.5 wt%).

### 2.3. Characterization

#### 2.3.1. Structure Characterization

The synthesized hydroxyapatite (HA) structure was characterized using X-ray diffraction (XRD) analysis conducted at the Advanced Characterization Laboratory 1, Physics-National Research and Innovation Agency. The XRD measurements were performed using a PANalytical AERIS instrument (Malvern, Worcestershire, United Kingdom) equipped with a copper (Cu) anode, voltage (40 kV), a PIXce11D-Medipix3 detector, and a monochromator with a *Kα_1_* wavelength of 1.540598 Å with an angle of 2θ from 5° to 85°. The obtained data were processed using Origin 8.5.1 (OriginLab, Northampton, MA, USA) and Rietica v4.2 software.

#### 2.3.2. Morphology Characterization

The morphology of the HA samples was analyzed using a scanning electron microscope (SEM) (JSM-6510LA) (JEOL, Akishima, Tokyo, Japan) with accelerated voltage (20 kV) at the Polymer Laboratory, National Research and Innovation Agency. Before imaging, the samples were coated with platinum (35 s; 30 mA) to enhance conductivity. SEM imaging was performed at magnifications ranging from 5000× to 15,000×. Furthermore, the captured SEM images were analyzed to determine the particle size distribution, average particle diameter, and material porosity using ImageJ 1.51 and Origin 8.5.1 (OriginLab, Northampton, USA) software [29].

#### 2.3.3. Physicochemical Properties

Physicochemical characteristics of HA samples synthesized using a porogen were examined using a digital pH meter (MP220 pH Meter, Mettler Toledo, Greifensee, Switzerland) and electrical conductometer (MC 226 Conductivity Meter, Mettler Toledo, Greifensee, Switzerland) [30].

## 3. Results

### 3.1. Hydroxyapatite Synthesis

Calcium oxide was successfully obtained with chicken eggshell as a source. The weight of chicken eggshells obtained before and after calcination was 98.7548 g and 65.5125 g; at a temperature of 750 °C, CaCO_3_ started to decompose into CaO and was completely decomposed at 1000 °C. Mass reduction was caused by the presence of water and organic compounds which evaporated in the calcination process [24]. The calcination reaction that occurred from the chicken eggshells which convert CaCO_3_ into CaO was based on Equation (2):

CaCO_(s)_ → CaO_(s)_ + CO_2(g)_ (2)

Hydroxyapatite (HA) powder was successfully synthesized using a hydrothermal method at a temperature of 230 °C for 48 h. The formation of HA in the hydrothermal synthesis process was based on the following reaction:

10 CaO_(s)_ + 6(NH_4_)2HPO_4(s)_ + 4H_2_O_(l)_ → Ca_10_(PO_4_)_6_(OH)_2_(s) + 12NH_4_OH_9(aq)_ (3)

The formation of the HA phase occurs gradually during the solvothermal reaction. Porous HA was formed as a result of modification with a polyvinyl alcohol (PVA) porogen, where PVA infiltrated into HA has a lower melting point compared to the densification temperature of HA. Therefore, during the calcination process at 1000 °C, PVA will vaporize and leave pores in HA [31,32]. 

### 3.2. Characterization

#### 3.2.1. Characterization of HA Structure Using XRD

**Figure 1:**
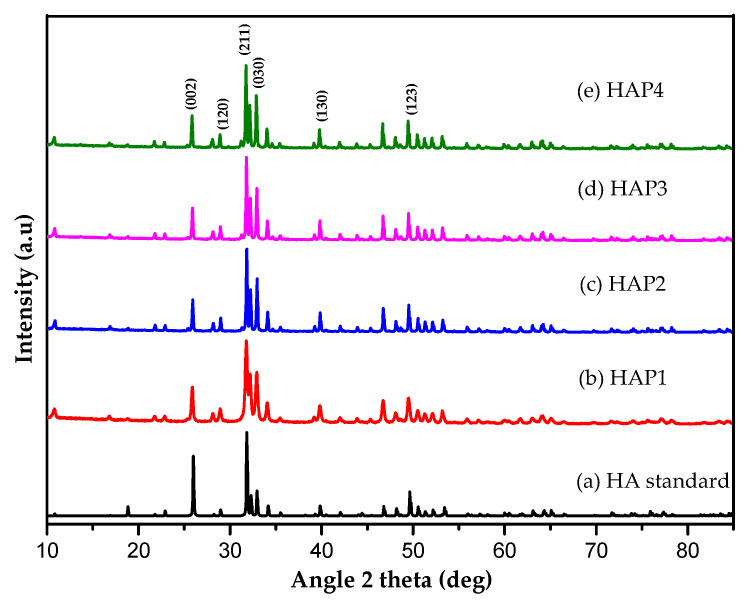
XRD pattern of: (**a**) HA standard (ICSD code 157481), (**b**) HAP1, (**c**) HAP2, (**d**) HAP3, and (**e**) HAP4.

Analysis of modified HA diffractograms with various concentrations of PVA has been conducted to determine the structure and phases of HA, followed by analysis using Origin 8.5.1 (OriginLab, Northampton, MA, USA) and Rietica v4.2 software. The diffraction patterns of the samples can be seen in Figure 1.

The XRD pattern of HA synthesized with PVA as a porogen is shown in Figure 1 and corresponds to the ICSD code 157481 standard with P*63/m* space group, number of space groups 176 (release 2007) [33], indicating the successful synthesis of hydroxyapatite. Based on Figure 1, the XRD pattern shows the typical peak of HA appears at 2θ: 25.87°; 28.9°; 31.77°; 32.80°; 39.72°; and 49.54° with the *hkl* of (002); (120); (211); (030); (130); and (123), and the highest peak in HA appears at 31.77° [34]. The presence of these peaks revealed that the HA was successfully synthesized using this method. The diffractogram pattern exhibits the same diffraction pattern as the HA synthesized without PVA as a porogen, and the highest HA peak for all variations of porogen addition was the same as the highest HA peak without a porogen. This indicates that all PVA porogens were removed from the HA during the calcination process, but there was a peak shift caused by changes in lattice parameters and lattice strain [10,21,35]. Based on the XRD pattern of HA without the addition of PVA porogen, HA can be indexed to the hexagonal HA phase (P*63/m* space group) with lattice parameters *a = b* = 9.412° and *c* = 6.853 Å (ICSD code 157481).

**Figure 2:**
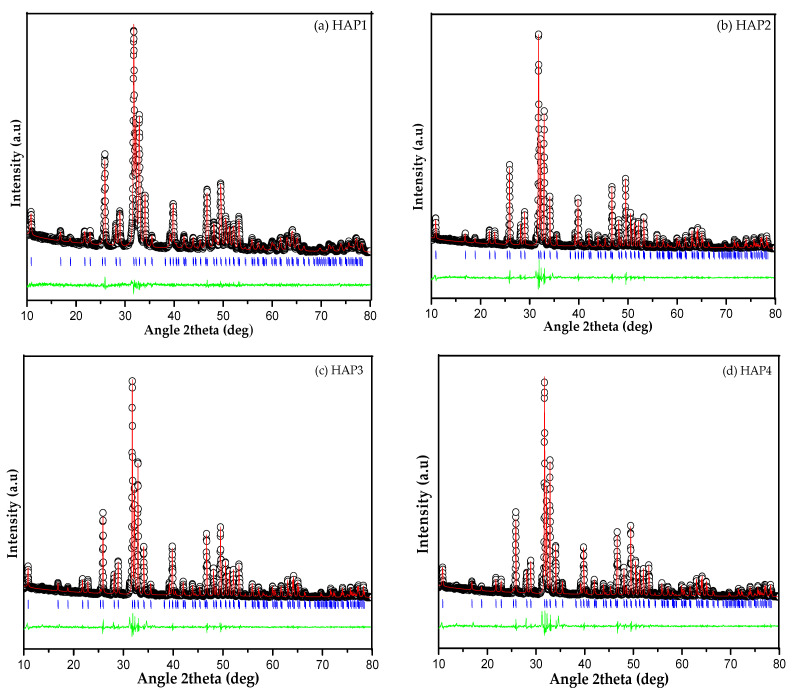
Le Bail refinement fit to SRD data for HA samples: (**a**) HAP1, (**b**) HAP2, (**c**) HAP3, and (**d**) HAP4. The black plot marks the experimentally observed pattern; the red plot marks the calculated diffraction pattern. The position of the Bragg peaks is marked with blue plot (vertical lines), and the difference between observed and calculated patterns is represented by the green plot at the bottom of the figure.

The synthesized compounds from the X-ray diffraction (XRD) pattern were analyzed using the *Le Bail* refinement method to study the phase formations and crystal structure in detail. The compound was analyzed using Rietica v4.2 software and the phase used for refinement in this study was the ICSD (Inorganic Crystal Structure Database) code 157481 standard of the structural data (release 2007), which has a P*63/m* space group with a space group number 176 with a hexagonal crystal structure and lattice parameters (*a* = *b =* 9.412 Å, and *c =* 6.853 Å). Refinement was carried out until a close fit between observed and calculated pattern was observed. Visual result of refined plots (Figure 2) showed the accomplishment of Le Bail refinement method and also showed a small difference between observed and calculated diffraction. It can be observed that the analysis result of the sample matches the standard, resulting in a red plot (calculated by the software) that aligns well with the black plot (sample), as evidenced by the blue plot (Bragg positions). The purity of the phase can be determined by the presence of blue lines following each peak. Following the use of the Le Bail refinement method for the XRD analysis of the obtained samples, indicating that a single phase of the formed compound is Ca_10_(PO_4_)_6_(OH)_2_ with a hexagonal structure and the P*63/m* space group without revealing another supplemented phase in the samples [36].

#### 3.2.2. Characterization of HA Morphology Using SEM

**Figure 3:**
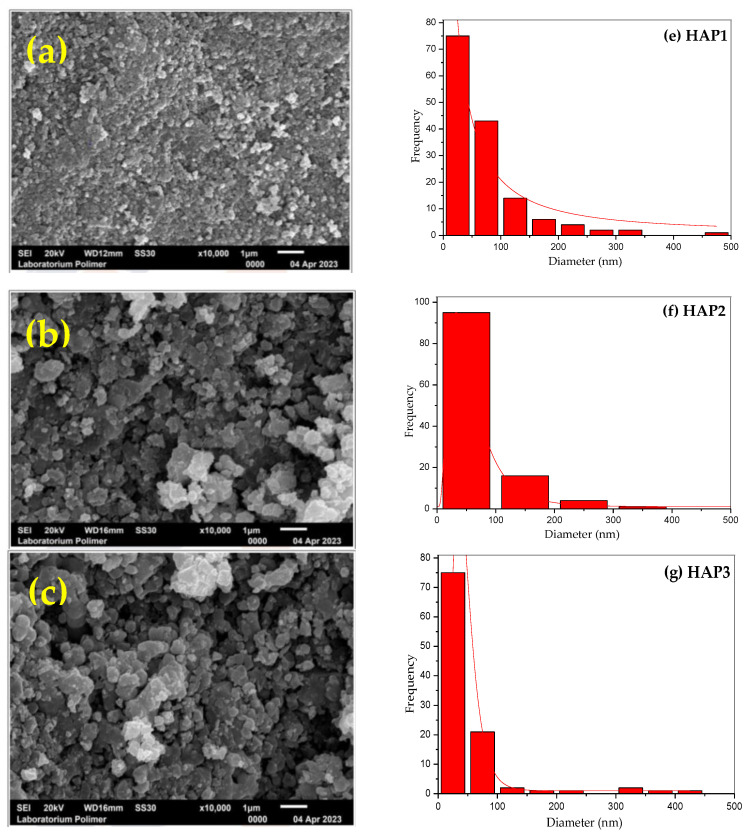

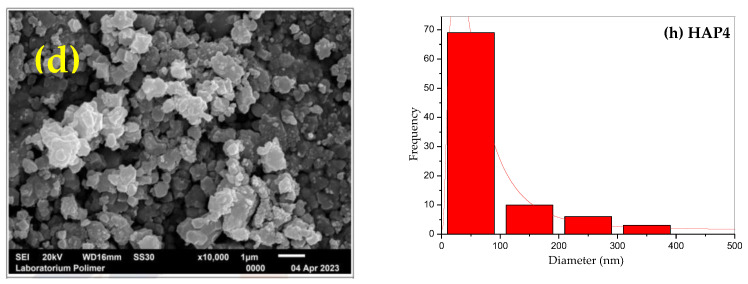
SEM image of HA samples: (**a**) HAP1, (**b**) HAP2, (**c**) HAP3, (**d**) HAP4 and particle size distribution of HA samples (**e**) HAP1, (**f**) HAP2, (**g**) HAP3, and (**h**) HAP4.

Morphological observations of hydroxyapatite (HA) samples were conducted using a scanning electron microscope (SEM) (JEOL, Akishima, Tokyo, Japan) for all samples (HAP1, HAP2, HAP3, and HAP4) at a magnification of 5000× to 15,000×. The average particle size and particle size distribution were analyzed using image analysis software called ImageJ 1.51 and Origin 8.5.1 (OriginLab, Northampton, MA, USA). In SEM images, HA was found in spherical shape, which differs from the previously obtained granular form of HA [21]. The difference could be attributed to differences in the precursor CaO source employed in the HA synthesis process and the morphology of HA differed from that of the modified HA, indicating that the calcination process released PVA and created voids, as can be seen in Figure 3. The morphology observations of HA revealed that the addition of PVA (2.5%, 5.0%, and 7.5% by *wt*) resulted in the formation of pores within the hydroxyapatite. These pores appeared as black spots or areas in the SEM images and the pores increased with increasing the concentration of PVA porogen [32]. The addition of PVA porogen also resulted in a more uniform particle distribution as can be seen in bar charts. As shown by analysis of the SEM results using ImageJ 1.51 software, modified HA with the PVA porogen had an average diameter particle of 68.08, 69.32, 50.63, and 86.92 nm, respectively, for each sample (HAP1, HAP2, HAP3, and HAP4). 

#### 3.2.3. Physicochemical Characteristics

The physicochemical characteristics of the synthesized samples are presented in Table 1, illustrating a correlation between increased porogen concentration and elevated pH values. Furthermore, as the pH shifts toward alkalinity, a concurrent improvement in electrical conductivity is observed, and the concentration of ions in alkaline solutions affects the value of electrical conductivity [37].

## 4. Discussion

The present result shows that polyvinyl alcohol (PVA) porogen use in hydroxyapatite (HA) synthesis influenced the characteristic of HA. The crystal size (D) and the lattice strain (ε) of the samples (HAP1, HAP2, HAP3, and HAP4) were changed by addition of PVA (0%, 2.5%, 5.0%, and 7.5% by wt), which caused a small shift peak in the XRD pattern of HA. The crystal size (D) was calculated based on the Scherrer Equation (4), and lattice strain (ε) of each sample was calculated based on Bragg’s law (5), which can be seen in Table 2 [38,39].

(4) $$D=\frac{Kʎ}{\beta \cos\theta }$$

(5) $$\varepsilon =\frac{\beta }{4\;\times \;\tan\theta }$$

where *K* is a constant (0.89), ʎ is the X-ray wavelength in nanometers (0.154 nm), β is the peak width of the diffraction peak at half maximum height, and the symbol θ can be degrees or radians where cos θ and tan θ refer to the θ values.

The average crystalline size of the HA samples was 46.06 nm. The crystalline size of the modified HA with addition of PVA porogen (0%, 2.5%, 5.0%, and 7.5% by *wt*) was increased, while a decrease of the lattice strain was observed with increasing PVA concentration and the shift peak of HAP2, HAP3, and HAP4 occurred from changes in the lattice strain of the HA samples. 

The Le Bail refinement analysis of the XRD pattern revealed changes in the unit cell parameters (lattice parameter and cell volume) of the HA sample, as shown in Table 3.

Table 3 shows that the unit cell parameters (space group, lattice parameter, cell volume), *R* factors (*Rp, Rwp*, and *Rexp*), and the goodness of fit (Chi square, χ^2^) of the HA sample analyzed using Le Bail refinement method, where the *GOF* (goodness of fit) which represented the χ^2^ value was calculated according to Equation (6) [40].

(6) GOF=Rwp/Rexp

The alterations in lattice parameters of the samples, as demonstrated in Table 3, exhibit slight deviations when contrasted with the ICSD code 157481 standard values (*a* = *b* = 9.412 Å, and *c* = 6.853 Å) [33]. These deviations, however, remain relatively inconspicuous despite the introduction of varying concentrations of PVA porogen and the influence of the sintering procedure. For the analyzed samples, an increase in the network parameter “a” and parameter “c” with increasing polyvinyl alcohol (PVA) concentration was observed. This finding was consistent with previous reports [41,42]. The correctness of the Le Bail refinement of the data obtained from the samples is monitored by a reliability index parameter such as the index of the pattern (*Rp*), the weighted pattern (*Rwp*), *Rexp*, and the index of χ, where the index of χ (goodness of fit) is given by the ratio of Rwp and *Rexp* factors. The obtained χ^2^ (Chi square) values were relatively low (< 4%) which was considered validating the acceptability of the data. In addition, the low values of the index of *R* factors (*Rp* and *Rwp*), below 20%, indicate a good agreement between the calculated data from the theoretical model and the observed pattern, thus validating the acceptance of the data [43].

**Figure 4:**
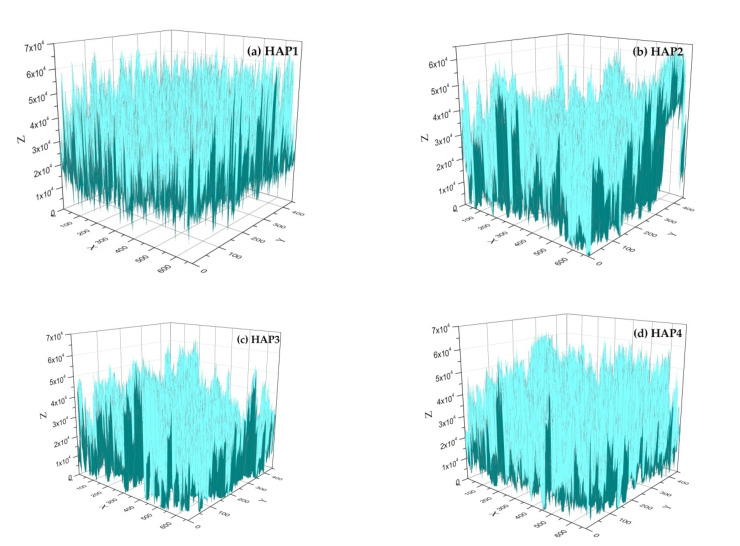
Three-dimensional graphics and porosity of the HA samples: (**a**) HAP1, (**b**) HAP2, (**c**) HAP3, and (**d**) HAP4.

The porosity of the HA material was analyzed and plotted in a 3D graph using Origin 8.5.1 software based on SEM images obtained and can be seen in Figure 4. This involves converting the image into a data format using the Origin software to generate a 2D matrix. The analysis of material volume was carried out in accordance with the application’s instructions. The volume of space beneath the sample surface, or the material pore volume, is determined using mathematical equations, while the total material volume is calculated based on the projected area of the sample surface in the x–y plane. Porosity is computed from the total volume and the volume of space beneath the surface. Finally, the data are visualized through a 3D surface projection [29]. 

It was evident that the blue colored area represents the solid volume of a material. In contrast, the white colored area represents the space/voids within the material. The addition of PVA 2.5 wt% resulted in a uniform pore distribution represented in blue and yielded a porosity of 67.10% (Figure 4b). The hydroxyapatite porosity increased to 69.53% with the supplemental PVA 5.0 wt%, the allocation of pores was still uniform, and the allocation of solid particles decreased (Figure 4c). By the supplemental PVA 7.5 wt%, the porosity decreased to 63.53%. Additionally, based on the image, it can be observed that with an increase in the porogen concentration from 2.5% to 7.5% by *wt*, the pores’ volume also increased compared to the volume of pores in HA without PVA porogen. This finding aligns with a previous report [32]. However, with the addition of 7.5 wt% PVA (HAP4), the porosity of the HA material decreased compared to concentrations of 2.5 wt% and 5.0 wt% (Figure 4b,c), although it still increased compared to HAP1 (Figure 4a).

The rise in porosity within HA material was linked to the introduction of the porogen. The incorporation of the porogen into the mixture caused a deceleration in the densification process, leading to an incomplete interlinkage among particles. This outcome is attributed to the presence of the porogen, which contributed to heightened porosity in the HA material due to the hindered densification process and incomplete particle interconnection. As a result, the porogen underwent evaporation upon the sample’s calcination, resulting in the creation of voids or gaps within the material. Thus, the porogen dissipated as the sample underwent calcination, leaving behind empty spaces within the material. This led to the formation of empty spaces within the material, as the porogen evaporated during the calcination process [19,21]. In this study, the HA material synthesized with a lower PVA concentration demonstrated higher porosity percentage compared to the earlier research conducted with a higher porogen concentration, incorporating PVA, paraffin wax and natural porogen from honeycomb [24,32,44]. 

Building upon the information provided, HAP samples modified with porogens yield materials with structure, morphologies, porosities, and physicochemical properties capable of creating a conducive environment for the growth of living organisms, facilitating the delivery of diverse biological molecules, and serving bone tissue engineering applications [24,26,45]. Nevertheless, achieving precise control and optimal alignment between porosity and physicochemical properties needs to be carried out for more specialized applications.

## 5. Conclusions

Hydroxyapatite (HA) has been successfully synthesized using a hydrothermal method using polyvinyl alcohol (PVA) as a porogenic agent. PVA is one of the porogens that influences the nanostructure of HA. Based on the characterization results of HA using X-ray diffraction (XRD), it was known that the resulting HA structure underwent changes in the unit cell parameters (lattice parameter and cell volumes), and based on refinement analysis, low chi square (χ^2^) and the index of R factors (*Rp* and *Rwp*) values indicate a good agreement between theoretical model and experiment pattern and validating the acceptability of the data. Morphological analysis of the sample using scanning electron microscope (SEM) revealed a more uniform particle distribution for PVA-modified HA than unmodified HA and the addition of PVA porogen resulted in the formation of pores. Additionally, the porosity of HA increased with the modification of HA using PVA porogen. The highest porosity was achieved with a modification of 5.0 wt% PVA porogen, reaching 69.53%. Materials possessing such properties can be utilized in carriers for delivering a diverse range of biological molecules or even as construction material and bone tissue engineering.

## Figures and Tables

**Table 1:** Physicochemical characteristics of HA.

Parameter	HAP1	HAP2	HAP3	HAP4
pH	8.95	9.89	9.85	9.79
EC (μs/cm)	55.4	80.50	81.45	64.15

The data represent the average of two replicates.

**Table 2:** Crystalline size and lattice strain of HA.

No.	Sample	Peak Position (°)	FWHM (Radian)	Crystalline Size (nm)	Lattice Strain
1.	HAP1	31.7729	0.2384	34.27	0.0369
2.	HAP2	31.8256	0.1631	50.10	0.0253
3.	HAP3	31.7988	0.1632	50.06	0.0252
4.	HAP4	31.7496	0.1640	49.79	0.0254

**Table 3:** Unit cell parameters of the samples were analyzed by the Le Bail refinement method.

No.	Cell Parameter	HAP1	HAP2	HAP3	HAP4
1.	*Space group*	P*63/m*	P*63/m*	P*63/m*	P*63/m*
2.	*a* = *b* (Å)	9.4316	9.4244	9.4222	9.4211
3.	*c* (Å)	6.8813	6.8832	6.8820	6.8814
4.	V (Å^3^)	530.1233	529.4619	529.1135	528.9485
5.	*Rp* (%)	4.21	6.07	6.17	6.89
6.	*Rwp* (%)	5.53	8.37	8.83	10.20
7.	*Rexp* (%)	4.14	4.23	4.25	4.20
8.	*χ^2^*	1.34	1.98	2.08	2.43

## Data Availability

Not applicable.
